# [(3*R*,4*S*)-4-(4-Fluoro­phen­yl)-1-methyl­piperidin-3-yl]methyl 4-methyl­benzene­sulfonate

**DOI:** 10.1107/S1600536810038249

**Published:** 2010-09-30

**Authors:** Jianfeng Qi, Hanjing Chen, Chen Zhang

**Affiliations:** a Department of Medicinal Chemistry, College of Pharmaceutical Science, Zhejiang University, Hangzhou 310058, People’s Republic of China

## Abstract

In the title compound, C_20_H_24_FNO_3_S, the piperidine ring adopts a chair conformation. The dihedral angle between the aromatic rings is 47.01 (17)°.

## Related literature

For general background to the design and synthesis of vinyl sulfonate derivatives, see: Curzons (2003 ▶), Segura *et al.* (2003 ▶). For related structures, see: Wang & Kanagawa (1997 ▶).
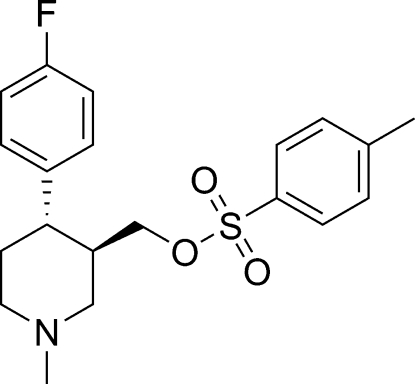


## Experimental

### 

#### Crystal data

C_20_H_24_FNO_3_S
*M* *_r_* = 377.46
Monoclinic, 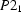

*a* = 9.1590 (4) Å
*b* = 10.0764 (5) Å
*c* = 10.7644 (6) Å
β = 95.718 (1)°
*V* = 988.50 (9) Å^3^
*Z* = 2
Mo *K*α radiation
μ = 0.19 mm^−1^
*T* = 296 K
0.32 × 0.26 × 0.20 mm

#### Data collection

Rigaku R-AXIS RAPID diffractometer
Absorption correction: multi-scan (*ABSCOR*; Higashi, 1995 ▶) *T*_min_ = 0.931, *T*_max_ = 0.963
9742 measured reflections
4457 independent reflections
3114 reflections with *I* > 2σ(*I*)
*R*_int_ = 0.021

#### Refinement

*R*[*F* ^2^ > 2σ(*F* ^2^)] = 0.031
*wR*(*F* ^2^) = 0.084
*S* = 1.00
4457 reflections
238 parameters
1 restraint
H-atom parameters constrained
Δρ_max_ = 0.17 e Å^−3^
Δρ_min_ = −0.14 e Å^−3^
Absolute structure: Flack (1983 ▶), 2086 Friedel pairs
Flack parameter: 0.05 (6)

### 

Data collection: *PROCESS-AUTO* (Rigaku, 2006 ▶); cell refinement: *PROCESS-AUTO*; data reduction: *CrystalStructure* (Rigaku/MSC, 2007 ▶); program(s) used to solve structure: *SHELXS97* (Sheldrick, 2008 ▶); program(s) used to refine structure: *SHELXL97* (Sheldrick, 2008 ▶); molecular graphics: *ORTEP-3 for Windows* (Farrugia, 1997 ▶); software used to prepare material for publication: *WinGX* (Farrugia, 1999 ▶).

## Supplementary Material

Crystal structure: contains datablocks global, I. DOI: 10.1107/S1600536810038249/kj2148sup1.cif

Structure factors: contains datablocks I. DOI: 10.1107/S1600536810038249/kj2148Isup2.hkl

Additional supplementary materials:  crystallographic information; 3D view; checkCIF report

## Figures and Tables

**Fig. 1.**
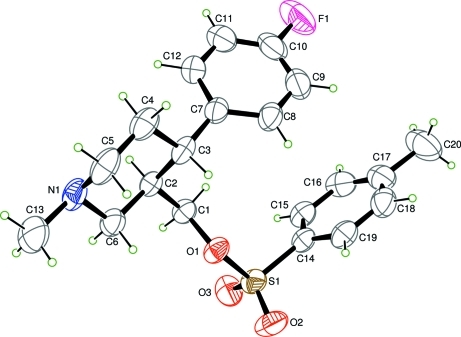
The molecular structure of the title compound showing atom labels and 40% probability displacement ellipsoids.

## supplementary crystallographic information

### Comment

The title compound is a useful intermediate in preparing paroxetine [(3*S*,4R)-4-(4-fluorophenyl)-3-(3,4-methylenedioxyphenoxymethyl)- piperidine]. Paroxetine is a well-known selective serotonin reuptake inhibitor (SSRI) antidepressant, used world wide in therapeutics (Segura *et al.*, 2003). In view of the above, ((3*R*,4S)-4-(4-fluorophenyl)-1-methylpiperidin-3-yl)methyl 4-methylbenzenesulfonate was synthesized and its crystal structure is reported here. A perspective view of the structure with the atomic numbering scheme is shown in Fig. 1. The dihedral angle between the two benzene rings is 47.01 (17)°. The piperidine ring adopts a chair conformation. The piperidine ring contains three planes (C2/C4/C5/C6, C3/C4/N1/C6, C2/C3/C5/N1), the first one of which is more planar than the other two.

### Experimental

To a stirred solution of trans-(-)-paroxo (10 g) in dichloromethane (50 ml) triethylamine (7 ml) was added. The mixture was cooled to 268 K. Toluenesulfonyl chloride (12 g) was slowly added and stirred for l h at 268 K. Methanesulfonic acid (4 ml) was then added gradually and the mixture was concentrated at about 323 K at atmospheric pressure. The residue was taken up in toluene, water was added and this was stirred for 30 minutes. The top toluene layer was separated. The pH of the aqueous layer was then adjusted to 9.0 with a saturated NaHCO_3_ solution. The product was filtered, washed with water and dried to yield the title compound (13 g) as a white to off-white solid (m.p. 380-381 K).

### Refinement

All H atoms were placed in calculated positions, with C—H distances in the range 0.93-0.98 and included in the final cycles of refinement in the riding-model approximation, with U_iso_(H) = 1.2U_eq_(C) or U_iso_(H) = 1.5U_eq_(C).

### Crystal data

**Table:**

C_20_H_24_FNO_3_S	*F*(000) = 400
*M**_r_* = 377.46	*D*_x_ = 1.268 Mg m^−^^3^
Monoclinic, *P*2_1_	Melting point: 380 K
Hall symbol: P 2yb	Mo *K*α radiation, λ = 0.71073 Å
*a* = 9.1590 (4) Å	Cell parameters from 7787 reflections
*b* = 10.0764 (5) Å	θ = 3.0–27.4°
*c* = 10.7644 (6) Å	µ = 0.19 mm^−^^1^
β = 95.718 (1)°	*T* = 296 K
*V* = 988.50 (9) Å^3^	Chunk, yellow
*Z* = 2	0.32 × 0.26 × 0.20 mm

### Data collection

**Table:**

Rigaku R-AXIS RAPID diffractometer	4457 independent reflections
Radiation source: rolling anode	3114 reflections with *I* > 2σ(*I*)
graphite	*R*_int_ = 0.021
Detector resolution: 10.00 pixels mm^-1^	θ_max_ = 27.4°, θ_min_ = 3.0°
ω scans	*h* = −11→11
Absorption correction: multi-scan (*ABSCOR*; Higashi, 1995)	*k* = −13→13
*T*_min_ = 0.931, *T*_max_ = 0.963	*l* = −13→13
9742 measured reflections	

### Refinement

**Table:**

Refinement on *F*^2^	Hydrogen site location: inferred from neighbouring sites
Least-squares matrix: full	H-atom parameters constrained
*R*[*F*^2^ > 2σ(*F*^2^)] = 0.031	*w* = 1/[σ^2^(*F*_o_^2^) + (0.0395*P*)^2^ + 0.063*P*] where *P* = (*F*_o_^2^ + 2*F*_c_^2^)/3
*wR*(*F*^2^) = 0.084	(Δ/σ)_max_ < 0.001
*S* = 1.00	Δρ_max_ = 0.17 e Å^−^^3^
4457 reflections	Δρ_min_ = −0.14 e Å^−^^3^
238 parameters	Extinction correction: *SHELXL97* (Sheldrick, 2008), Fc^*^=kFc[1+0.001xFc^2^λ^3^/sin(2θ)]^-1/4^
1 restraint	Extinction coefficient: 0.0052 (13)
Primary atom site location: structure-invariant direct methods	Absolute structure: Flack (1983), 2086 Friedel pairs
Secondary atom site location: difference Fourier map	Flack parameter: 0.05 (6)

### Special details

**Table:**

Geometry. All esds (except the esd in the dihedral angle between two l.s. planes) are estimated using the full covariance matrix. The cell esds are taken into account individually in the estimation of esds in distances, angles and torsion angles; correlations between esds in cell parameters are only used when they are defined by crystal symmetry. An approximate (isotropic) treatment of cell esds is used for estimating esds involving l.s. planes.
Refinement. Refinement of *F*^2^ against ALL reflections. The weighted *R*-factor wR and goodness of fit *S* are based on *F*^2^, conventional *R*-factors *R* are based on *F*, with *F* set to zero for negative *F*^2^. The threshold expression of *F*^2^ > σ(*F*^2^) is used only for calculating *R*-factors(gt) etc. and is not relevant to the choice of reflections for refinement. *R*-factors based on *F*^2^ are statistically about twice as large as those based on *F*, and *R*- factors based on ALL data will be even larger.

### Fractional atomic coordinates and isotropic or equivalent isotropic displacement parameters (Å^2^)

**Table:**

	*x*	*y*	*z*	*U*_iso_*/*U*_eq_	
S1	0.34071 (5)	0.65560 (6)	0.19254 (4)	0.05870 (14)	
O1	0.37947 (12)	0.66249 (15)	0.33871 (9)	0.0550 (3)	
C14	0.51158 (17)	0.6533 (2)	0.13267 (13)	0.0496 (4)	
O2	0.2721 (2)	0.52973 (18)	0.17277 (14)	0.0832 (5)	
C2	0.4586 (2)	0.77398 (17)	0.53214 (15)	0.0481 (4)	
H2	0.4844	0.8619	0.5665	0.058*	
O3	0.26443 (18)	0.77282 (18)	0.15090 (14)	0.0788 (5)	
N1	0.3547 (2)	0.71908 (16)	0.72847 (16)	0.0659 (5)	
C3	0.5921 (2)	0.68346 (16)	0.56744 (16)	0.0533 (5)	
H3	0.5703	0.5965	0.5292	0.064*	
C7	0.7277 (2)	0.73680 (18)	0.51511 (18)	0.0568 (5)	
C4	0.6116 (2)	0.6639 (3)	0.70958 (16)	0.0684 (5)	
H4A	0.6855	0.5963	0.7303	0.082*	
H4B	0.6463	0.7460	0.7492	0.082*	
C1	0.4245 (2)	0.79152 (17)	0.39340 (16)	0.0507 (4)	
H1A	0.3462	0.8558	0.3764	0.061*	
H1B	0.5105	0.8238	0.3571	0.061*	
C15	0.5673 (2)	0.7700 (2)	0.08678 (18)	0.0595 (5)	
H15	0.5152	0.8491	0.0888	0.071*	
C6	0.3242 (2)	0.7278 (2)	0.59338 (18)	0.0615 (5)	
H6A	0.2441	0.7895	0.5729	0.074*	
H6B	0.2937	0.6414	0.5604	0.074*	
C5	0.4712 (3)	0.62325 (19)	0.75996 (19)	0.0737 (7)	
H5A	0.4409	0.5373	0.7260	0.088*	
H5B	0.4878	0.6148	0.8500	0.088*	
C8	0.7843 (2)	0.6761 (3)	0.41456 (18)	0.0696 (6)	
H8	0.7424	0.5975	0.3828	0.083*	
C19	0.5895 (3)	0.5365 (2)	0.12864 (18)	0.0630 (5)	
H19	0.5529	0.4583	0.1595	0.076*	
C9	0.9024 (3)	0.7304 (3)	0.3602 (2)	0.0862 (7)	
H9	0.9399	0.6887	0.2931	0.103*	
C17	0.7790 (2)	0.6507 (4)	0.03166 (18)	0.0795 (6)	
C18	0.7223 (3)	0.5368 (3)	0.0783 (2)	0.0792 (7)	
H18	0.7748	0.4580	0.0759	0.095*	
C20	0.9241 (3)	0.6477 (6)	−0.0255 (3)	0.1361 (12)	
H20A	0.9107	0.6047	−0.1054	0.204*	
H20B	0.9953	0.5998	0.0285	0.204*	
H20C	0.9579	0.7369	−0.0357	0.204*	
F1	1.07463 (15)	0.9031 (2)	0.35259 (18)	0.1293 (7)	
C13	0.2204 (3)	0.6809 (4)	0.7843 (2)	0.1021 (10)	
H13A	0.1910	0.5934	0.7567	0.153*	
H13B	0.1435	0.7428	0.7590	0.153*	
H13C	0.2392	0.6815	0.8737	0.153*	
C12	0.7948 (2)	0.8534 (2)	0.5606 (2)	0.0732 (6)	
H12	0.7603	0.8954	0.6288	0.088*	
C10	0.9617 (2)	0.8460 (3)	0.4074 (3)	0.0858 (7)	
C16	0.7006 (3)	0.7662 (3)	0.0384 (2)	0.0751 (7)	
H16	0.7389	0.8444	0.0092	0.090*	
C11	0.9115 (3)	0.9079 (3)	0.5067 (3)	0.0885 (7)	
H11	0.9551	0.9859	0.5379	0.106*	

### Atomic displacement parameters (Å^2^)

**Table:**

	*U*^11^	*U*^22^	*U*^33^	*U*^12^	*U*^13^	*U*^23^
S1	0.0645 (3)	0.0783 (3)	0.0327 (2)	−0.0102 (3)	0.00190 (16)	0.0008 (3)
O1	0.0736 (7)	0.0586 (6)	0.0328 (5)	−0.0118 (8)	0.0056 (5)	0.0006 (7)
C14	0.0649 (9)	0.0540 (9)	0.0294 (7)	−0.0033 (11)	0.0024 (6)	0.0024 (9)
O2	0.0960 (12)	0.1015 (12)	0.0521 (9)	−0.0489 (10)	0.0078 (8)	−0.0117 (8)
C2	0.0602 (10)	0.0463 (9)	0.0372 (9)	−0.0004 (8)	0.0026 (7)	−0.0028 (7)
O3	0.0729 (10)	0.1132 (13)	0.0492 (9)	0.0227 (9)	0.0005 (7)	0.0127 (9)
N1	0.0924 (13)	0.0687 (10)	0.0391 (9)	−0.0094 (9)	0.0187 (8)	−0.0068 (7)
C3	0.0747 (11)	0.0445 (11)	0.0392 (9)	0.0079 (9)	−0.0017 (8)	−0.0071 (7)
C7	0.0597 (11)	0.0611 (11)	0.0475 (11)	0.0165 (9)	−0.0053 (9)	−0.0066 (9)
C4	0.0977 (13)	0.0628 (10)	0.0419 (9)	0.0124 (14)	−0.0073 (9)	0.0007 (11)
C1	0.0588 (10)	0.0501 (10)	0.0429 (10)	−0.0019 (8)	0.0036 (8)	0.0031 (7)
C15	0.0787 (14)	0.0583 (11)	0.0409 (10)	−0.0056 (10)	0.0027 (9)	0.0054 (9)
C6	0.0715 (13)	0.0689 (11)	0.0452 (11)	−0.0076 (10)	0.0106 (9)	−0.0042 (8)
C5	0.1250 (19)	0.0585 (15)	0.0376 (10)	−0.0056 (12)	0.0081 (11)	0.0020 (8)
C8	0.0716 (12)	0.0842 (15)	0.0506 (11)	0.0210 (13)	−0.0051 (9)	−0.0139 (12)
C19	0.0824 (15)	0.0587 (12)	0.0467 (12)	−0.0019 (11)	0.0004 (10)	−0.0012 (9)
C9	0.0689 (14)	0.130 (2)	0.0600 (15)	0.0322 (14)	0.0061 (11)	−0.0080 (13)
C17	0.0626 (11)	0.1280 (19)	0.0472 (11)	−0.0052 (18)	0.0019 (8)	−0.0036 (16)
C18	0.0810 (17)	0.0940 (17)	0.0603 (14)	0.0232 (14)	−0.0049 (12)	−0.0148 (13)
C20	0.0691 (14)	0.253 (4)	0.0883 (19)	−0.003 (3)	0.0194 (13)	−0.009 (3)
F1	0.0667 (8)	0.1822 (18)	0.1440 (16)	0.0034 (10)	0.0364 (9)	0.0170 (13)
C13	0.122 (2)	0.131 (3)	0.0591 (14)	−0.040 (2)	0.0382 (13)	−0.0086 (15)
C12	0.0656 (12)	0.0774 (14)	0.0771 (15)	0.0049 (11)	0.0098 (11)	−0.0234 (11)
C10	0.0482 (12)	0.118 (2)	0.0909 (19)	0.0140 (13)	0.0073 (12)	0.0047 (16)
C16	0.0785 (16)	0.0988 (18)	0.0482 (13)	−0.0271 (14)	0.0079 (11)	0.0101 (12)
C11	0.0601 (13)	0.0933 (17)	0.112 (2)	−0.0020 (12)	0.0077 (13)	−0.0169 (15)

### Geometric parameters (Å, °)

**Table:**

S1—O3	1.4219 (18)	C6—H6A	0.9700
S1—O2	1.4220 (17)	C6—H6B	0.9700
S1—O1	1.5796 (10)	C5—H5A	0.9700
S1—C14	1.7510 (16)	C5—H5B	0.9700
O1—C1	1.469 (2)	C8—C9	1.392 (3)
C14—C19	1.379 (3)	C8—H8	0.9300
C14—C15	1.392 (3)	C19—C18	1.380 (4)
C2—C1	1.505 (2)	C19—H19	0.9300
C2—C6	1.527 (3)	C9—C10	1.362 (4)
C2—C3	1.542 (2)	C9—H9	0.9300
C2—H2	0.9800	C17—C16	1.374 (4)
N1—C5	1.454 (3)	C17—C18	1.375 (4)
N1—C6	1.456 (2)	C17—C20	1.519 (3)
N1—C13	1.474 (3)	C18—H18	0.9300
C3—C7	1.513 (3)	C20—H20A	0.9600
C3—C4	1.535 (2)	C20—H20B	0.9600
C3—H3	0.9800	C20—H20C	0.9600
C7—C8	1.387 (3)	F1—C10	1.368 (3)
C7—C12	1.392 (3)	C13—H13A	0.9600
C4—C5	1.501 (3)	C13—H13B	0.9600
C4—H4A	0.9700	C13—H13C	0.9600
C4—H4B	0.9700	C12—C11	1.380 (3)
C1—H1A	0.9700	C12—H12	0.9300
C1—H1B	0.9700	C10—C11	1.356 (4)
C15—C16	1.375 (3)	C16—H16	0.9300
C15—H15	0.9300	C11—H11	0.9300
			
O3—S1—O2	119.85 (9)	C2—C6—H6B	109.3
O3—S1—O1	109.40 (9)	H6A—C6—H6B	108.0
O2—S1—O1	103.91 (9)	N1—C5—C4	111.70 (17)
O3—S1—C14	108.92 (10)	N1—C5—H5A	109.3
O2—S1—C14	109.32 (12)	C4—C5—H5A	109.3
O1—S1—C14	104.29 (6)	N1—C5—H5B	109.3
C1—O1—S1	117.67 (11)	C4—C5—H5B	109.3
C19—C14—C15	120.08 (17)	H5A—C5—H5B	107.9
C19—C14—S1	120.46 (16)	C7—C8—C9	121.4 (2)
C15—C14—S1	119.45 (17)	C7—C8—H8	119.3
C1—C2—C6	111.55 (15)	C9—C8—H8	119.3
C1—C2—C3	113.27 (14)	C14—C19—C18	119.3 (2)
C6—C2—C3	111.54 (15)	C14—C19—H19	120.4
C1—C2—H2	106.7	C18—C19—H19	120.4
C6—C2—H2	106.7	C10—C9—C8	118.5 (2)
C3—C2—H2	106.7	C10—C9—H9	120.8
C5—N1—C6	109.66 (15)	C8—C9—H9	120.8
C5—N1—C13	110.76 (19)	C16—C17—C18	117.99 (19)
C6—N1—C13	109.76 (18)	C16—C17—C20	121.4 (3)
C7—C3—C4	113.49 (15)	C18—C17—C20	120.6 (4)
C7—C3—C2	110.99 (14)	C17—C18—C19	121.7 (2)
C4—C3—C2	109.40 (15)	C17—C18—H18	119.2
C7—C3—H3	107.6	C19—C18—H18	119.2
C4—C3—H3	107.6	C17—C20—H20A	109.5
C2—C3—H3	107.6	C17—C20—H20B	109.5
C8—C7—C12	117.4 (2)	H20A—C20—H20B	109.5
C8—C7—C3	121.21 (18)	C17—C20—H20C	109.5
C12—C7—C3	121.27 (18)	H20A—C20—H20C	109.5
C5—C4—C3	112.10 (16)	H20B—C20—H20C	109.5
C5—C4—H4A	109.2	N1—C13—H13A	109.5
C3—C4—H4A	109.2	N1—C13—H13B	109.5
C5—C4—H4B	109.2	H13A—C13—H13B	109.5
C3—C4—H4B	109.2	N1—C13—H13C	109.5
H4A—C4—H4B	107.9	H13A—C13—H13C	109.5
O1—C1—C2	108.42 (13)	H13B—C13—H13C	109.5
O1—C1—H1A	110.0	C11—C12—C7	121.4 (2)
C2—C1—H1A	110.0	C11—C12—H12	119.3
O1—C1—H1B	110.0	C7—C12—H12	119.3
C2—C1—H1B	110.0	C11—C10—C9	122.2 (2)
H1A—C1—H1B	108.4	C11—C10—F1	118.5 (3)
C16—C15—C14	118.8 (2)	C9—C10—F1	119.3 (3)
C16—C15—H15	120.6	C17—C16—C15	122.2 (2)
C14—C15—H15	120.6	C17—C16—H16	118.9
N1—C6—C2	111.50 (16)	C15—C16—H16	118.9
N1—C6—H6A	109.3	C10—C11—C12	119.1 (2)
C2—C6—H6A	109.3	C10—C11—H11	120.4
N1—C6—H6B	109.3	C12—C11—H11	120.4
			
O3—S1—O1—C1	−38.54 (14)	C13—N1—C6—C2	177.1 (2)
O2—S1—O1—C1	−167.66 (14)	C1—C2—C6—N1	−176.31 (15)
C14—S1—O1—C1	77.84 (14)	C3—C2—C6—N1	55.9 (2)
O3—S1—C14—C19	−161.67 (15)	C6—N1—C5—C4	61.7 (2)
O2—S1—C14—C19	−29.00 (16)	C13—N1—C5—C4	−177.04 (18)
O1—S1—C14—C19	81.61 (16)	C3—C4—C5—N1	−57.2 (2)
O3—S1—C14—C15	17.09 (17)	C12—C7—C8—C9	−0.8 (3)
O2—S1—C14—C15	149.76 (15)	C3—C7—C8—C9	175.15 (18)
O1—S1—C14—C15	−99.62 (15)	C15—C14—C19—C18	−0.3 (3)
C1—C2—C3—C7	57.92 (18)	S1—C14—C19—C18	178.51 (16)
C6—C2—C3—C7	−175.24 (14)	C7—C8—C9—C10	−0.4 (3)
C1—C2—C3—C4	−176.09 (16)	C16—C17—C18—C19	1.1 (3)
C6—C2—C3—C4	−49.2 (2)	C20—C17—C18—C19	−178.8 (2)
C4—C3—C7—C8	129.5 (2)	C14—C19—C18—C17	−0.2 (3)
C2—C3—C7—C8	−106.83 (19)	C8—C7—C12—C11	1.1 (3)
C4—C3—C7—C12	−54.7 (2)	C3—C7—C12—C11	−174.8 (2)
C2—C3—C7—C12	68.9 (2)	C8—C9—C10—C11	1.3 (4)
C7—C3—C4—C5	174.59 (17)	C8—C9—C10—F1	−177.8 (2)
C2—C3—C4—C5	50.0 (2)	C18—C17—C16—C15	−1.7 (3)
S1—O1—C1—C2	−178.87 (11)	C20—C17—C16—C15	178.3 (2)
C6—C2—C1—O1	−61.63 (18)	C14—C15—C16—C17	1.3 (3)
C3—C2—C1—O1	65.21 (18)	C9—C10—C11—C12	−1.1 (4)
C19—C14—C15—C16	−0.3 (3)	F1—C10—C11—C12	178.1 (2)
S1—C14—C15—C16	−179.04 (15)	C7—C12—C11—C10	−0.2 (4)
C5—N1—C6—C2	−61.0 (2)		
